# Comprehensive sensory and chemical data on the flavor of 16 red wines from two varieties: Sensory descriptive analysis, HS-SPME-GC-MS volatile compounds quantitative analysis, and odor-active compounds identification by HS-SPME-GC-MS-O

**DOI:** 10.1016/j.dib.2019.103725

**Published:** 2019-03-06

**Authors:** Angélique Villière, Ronan Symoneaux, Alice Roche, Aïda Eslami, Nathalie Perrot, Yves Le Fur, Carole Prost, Philippe Courcoux, Evelyne Vigneau, Thierry Thomas-Danguin, Laurence Guérin

**Affiliations:** a ONIRIS, Nantes-Atlantic College of Veterinary Medicine and Food Science, UMR GEPEA CNRS 6144, BP 82225, F-44307, Nantes, France; b USC 1422 GRAPPE, INRA, Ecole Supérieure D'Agricultures, Univ. Bretagne Loire, SFR 4207 QUASAV, SensoVeg, F-49100 Angers, France; c Centre des Sciences du Goût et de L'Alimentation, UMR1324 INRA, UMR6265 CNRS, AgroSup Dijon, Université de Bourgogne Franche – Comté, F-21000 Dijon, France; d StatSC, Oniris, INRA, 44322 Nantes, France; e Univ Paris Saclay, UMR GMPA, AgroParisTech, INRA, F-78850 Thiverval Grignon, France; f Inst Francais Vigne & Vin, Unite Vins Innovat Itineraires Terroirs & Acteurs, Amboise, France

**Keywords:** Wine, Descriptive sensory analysis, VOC, Olfactometry, GC-MS-O

## Abstract

This paper describes data collected on 2 sets of 8 French red wines from two grape varieties: Pinot Noir (PN) and Cabernet Franc (CF). It provides, for the 16 wines, (i) sensory descriptive data obtained with a trained panel, (ii) volatile organic compounds (VOC) quantification data obtained by Headspace Solid Phase Micro-Extraction – Gas Chromatography – Mass Spectrometry (HS-SPME-GC-MS) and (iii) odor-active compounds identification by Headspace Solid Phase Micro-Extraction – Gas Chromatography – Mass Spectrometry – Olfactometry (HS-SPME-GC-MS-O). The raw data are hosted on an open-access research data repository [1].

**Table:** Specifications table

Subject area	*Food science*
More specific subject area	*Wine flavor research*
Type of data	*Microsoft Excel Worksheet containing 8 sheets: (1) Information, (2) Experimental factors, (3) List sensory descriptors, (4) Sensory descriptive analysis, (5) List VOC, (6) VOC quantification, (7) List GC-MS-O and (8) GC-MS-O*
How data was acquired	- Sensory descriptive analysis: The intensity of 33 sensory descriptors was rated by 16 trained panelists - VOC quantification: Volatile compounds in wines were extracted using Headspace Solid Phase Micro-Extraction (HS-SPME) and analyzed with Gas Chromatography coupled with Mass Spectrometry (GC-MS) - Odor-active compounds: Odor-active compounds were identified using Gas Chromatography coupled with Mass Spectrometry and Olfactometry (GC-MS-O) after Headspace Solid Phase Micro-Extraction (HS-SPME). Eight GC-MS-O analyses were carried out for each 16 wines.
Data format	*Table in raw format (.xlsx)*[1]
Experimental factors	*The experimental factors were: the grape variety, the vintage and the Protected Designation of Origin (PDO) of the wines*
Experimental features	- Sensory descriptive analysis: Sensory odor profile of the wines - VOC quantification: Quantitative data (μg.L^−1^ in headspace) on the volatile compounds in the wines - Odor-active compounds: Odor-active compounds among the VOCs found in the wines, their detection by 8 panelists and their odor characteristics
Data source location	- Descriptive sensory data were obtained at USC 1422 GRAPPE, INRA, Ecole Supérieure d’Agricultures, Univ. Bretagne Loire, SFR 4207 QUASAV, SensoVeg, F-49100 Angers, France - GCO data were obtained at ONIRIS, Nantes-Atlantic College of Veterinary Medicine and Food Science, UMR GEPEA CNRS 6144, BP 82225, F-44307, Nantes, France
Data accessibility	The raw data, provided as a Microsoft Excel Worksheet, are available on the Zenodo open-access research data repository [1], http://doi.org/10.5281/zenodo.1213610
Related research article	Roche, A., Perrot, N., Chabin, T., Villière, A., Symoneaux, R., Thomas-Danguin, T. (2017, May). In silico modelling to predict the odor profile of food from its molecular composition using experts' knowledge, fuzzy logic and optimization: Application on wines. In ISOCS/IEEE International Symposium on Olfaction and Electronic Nose (ISOEN) pp. 1–3. http://doi.org/10.1109/ISOEN.2017.7968875

**Table:** 

**Value of the data** • The data can help researchers to link sensory qualities of wines to their chemical composition [2]. • The data can be used along with other datasets as a benchmark to develop methods and tools to predict the odor of wines [3]. • The data can be compared to other wines varying in grape variety and vintage.

## 1 Data

The dataset gathered, for the 16 wines from two grape varieties, 4 blocks of data: (1) the experimental factors (the grape variety, the vintage and the Protected Designation of Origin; Table 1), (2) the sensory descriptive data obtained with a trained panel using 33 sensory descriptors (Table 2), (3) the volatile organic compounds (VOC) quantification data obtained for 45 target odorants by Headspace Solid Phase Micro-Extraction – Gas Chromatography – Mass Spectrometry (Table 3) and (4) the odor-active compounds, identified by Headspace Solid Phase Micro-Extraction – Gas Chromatography – Mass Spectrometry – Olfactometry (Table 4).

**Table 1:** Wines experimental factors.

Wine	Grape_variety	Vintage	PDO
PN1	Pinot Noir	2010	Bourgogne
PN2	Pinot Noir	2009	Bourgogne
PN3	Pinot Noir	2009	Bourgogne
PN4	Pinot Noir	2009	Bourgogne Hautes Côtes de Beaune
PN5	Pinot Noir	2009	Savigny-lès-Beaune
PN6	Pinot Noir	2010	Maranges
PN7	Pinot Noir	2009	Côte de Nuits-Villages
PN8	Pinot Noir	2009	Ladoix
CF1	Cabernet Franc	2010	Bourgueil
CF2	Cabernet Franc	2010	Chinon
CF3	Cabernet Franc	2009	Chinon
CF4	Cabernet Franc	2010	St-Nicolas-de-Bourgueil
CF5	Cabernet Franc	2010	Bourgueil
CF6	Cabernet Franc	2010	Bourgueil
CF7	Cabernet Franc	2010	Bourgueil
CF8	Cabernet Franc	2010	Saumur

**Table 2:** Sensory descriptors used by the trained panel for the sensory descriptive analysis.

Artichoke	Clove	Plum fresh
Bell pepper	Cut grass	Prune
Blackberry fresh	Elderflower	Raspberry fresh
Blackcurrant bud	Ethanol	Smoky
Blackcurrant fresh	Firestone	Strawberry cooked
Blueberry fresh	Geranium	Strawberry fresh
Brioche	Hay	Toasty
Butter	Leather	Undergrowth
Cherry cooked	Musk	Vanilla
Cherry fresh	Pepper	Violet
Cherry stone	Plum cooked	Woody

**Table 3:** Volatile organic compounds (VOC) quantified by GC-MS analysis and their corresponding CAS number.

VOC	CAS number
1-Hexanol	111-27-3
1-Octanol	111-87-5
1-Phenoxy-2-propanol	770-35-4
2,3-Butanedione	431-03-8
2-Ethylhexan-1-ol	104-76-7
2-Isobutyl-3-methoxypyrazine	24683-00-9
2-Methyl-1-butanol	137-32-6
2-Methylbutyl acetate	624-41-9
2-Phenylethanol	60-12-8
3-Methyl-1-butanol	123-51-3
4-Ethyl-2-methoxyphenol	2785-89-9
4-Ethylphenol	123-07-9
Acetaldehyde	75-07-0
Acetic acid	64-19-7
alpha-Ionone	127-41-3
Beta-Ionone	79-77-6
Butyl acetate	123-86-4
Butyric acid	107-92-6
Damascenone	23726-93-4
Dimethyl Sulfide	75-18-3
Ethyl 2-methylbutyrate	7452-79-1
Ethyl 3-hydroxybutyrate	5405-41-4
Ethyl 6-hydroxyhexanoate	5299-60-5
Ethyl acetate	141-78-6
Ethyl butyrate	105-54-4
Ethyl caproate	123-66-0
Ethyl isobutyrate	97-62-1
Ethyl isovalerate	108-64-5
Ethyl lactate	97-64-3
Ethyl octanoate	106-32-1
Ethyl propionate	105-37-3
Furaneol	3658-77-3
Hexyl acetate	142-92-7
Homofuraneol	27538-10-9
Isoamyl acetate	123-92-2
Isoamyl propionate	105-68-0
Isovaleric acid	503-74-2
Methional	3268-49-3
Methionol	505-10-2
Pentyl propionate	624-54-4
Phenol	108-95-2
Phenylacetaldehyde	122-78-1
Phenylacetic acid	103-82-2
Propionic acid	79-09-4
trans-3-Hexen-1-ol	544-12-7

**Table 4:** Linear Retention Index (apex) of odorant zones detected in GC-MS-O analysis of the wines, the name of the corresponding identified compounds and their CAS numbers. Compounds that appear in italics were tentatively identified owing to MS spectra, odor quality and LRI but available data could not allow discriminating between isomers.

LRI	Odorant	CAS
1309	1-Octen-3-one	4312-99-6
979	2,3-Butanedione	431-3-8
1063	2,3-Pentanedione	600-14-6
2270	2,6-Dimethoxyphenol	91-10-1
1877	2-Methoxyphenol	90-05-1
1020	2-Methylpropyl acetate	110-19-0
1540	3-Isobutyl-2-methoxypyrazine	24683-00-9
1437	3-Isopropyl-2-methoxypyrazine	25773-40-4
1854	3-Mercapto-1-hexanol	51755-83-0
1216	3-Methyl-1-butanol	123-51-3
927	3-Methylbutanal	590-86-3
1134	3-Methylbutyl acetate	123-92-2
2039	4-Ethyl guaïacol	2785-89-9
*2179*	*4 (or 3)-Ethylphenol*	*123-07-9 (or 620-17-7)*
1321	4-Methyl-1-pentanol	626-89-1
715	Acetaldehyde	75-07-0
1450	Acetic acid	64-19-7
1561	Benzaldehyde	100-52-7
1666	Benzene acetaldehyde	122-78-1
1926	Benzene ethanol	60-12-8
1902	Benzene methanol	100-51-6
1632	Butyric acid	107-92-6
1666	Butyrolactone	96-48-0
764	Dimethyl sulfide	75-18-3
942	Ethanol	64-17-5
914	Ethyl acetate	141-78-6
1046	Ethyl butanoate	105-54-4
1846	Ethyl dodecanoate	106-33-2
1241	Ethyl hexanoate	123-66-0
1437	Ethyl octanoate	106-32-1
964	Ethyl propanoate	105-37-3
1061	Ethyl-2-methylbutanoate	7452-79-1
970	Ethyl-2-methylpropanoate	97-62-1
1076	Ethyl-3-methylbutanoate	108-64-5
1671	Isovaleric acid	503-74-2
700	Methanethiol	74-93-1
1470	Methional	3268-49-3
1729	Methionol	505-10-2
1017	Methyl-2-methylpropenoate	80-62-6
*2080*	*p (or m)-Cresol*	*106-44-5 (or 108-39-4)*
1828	Phenethyl acetate	103-45-7
1998	Phenol	108-95-2
867	Sulphur dioxide	7446-09-5
1987	Whyskeylactone	39212-23-2

## 2 Experimental design, materials, and methods

### 2.1 Wines

Two sets of French red wines from two grape varieties, 8 Pinot Noir wines (PN) and 8 Cabernet Franc wines (CF) were analyzed (Table 1). The wines were selected out of 40 wines previously studied [4]. The main factors allowed for were vintage (2009 and 2010) and Protected Designation of Origin (PDO).

### 2.2 Sensory descriptive analysis

The sensory descriptive analysis of the 16 wines was performed at Groupe ESA, USC GRAPPE Senso’Veg (Angers, France).

#### 2.2.1 Wines preparation

The wines were opened 30 minutes before the sensory evaluation and served (5 cL) in white ISO wine tasting glasses [5] at room temperature.

#### 2.2.2 Sensory evaluation

Sixteen trained panelists, 6 women and 10 men (age range 35–71), participated in the sensory sessions. Sensory evaluation was performed according to recommended practices [6]. Before the sensory descriptive experiment, the judges were trained in 17 training sessions of 1-h each. This training consisted in a familiarization with the task and with the vocabulary and a selection of specific sensory descriptors for the wines set. During the familiarization step, the panelists did odor recognition tests on testing strip and on wines to become familiar with the sensory descriptors used for wines and smelled different standard odor references. These reference standards were adapted from [7]. During the sensory descriptors selection, the panelists were provided with an initial list of 84 descriptors. The list was elaborated by compiling terms from other lists employed in the description of wines from different varieties and geographical origins. Descriptors were arranged in the list by odor families: animal, burnt, floral, fruity, herbaceous, mineral, nut, spicy, undergrowth and others. Panelists modified the initial list of terms by removing those terms they considered irrelevant, ambiguous or redundant and by adding new attributes they considered pertinent while describing 15 wines of similar characteristics (grape variety and origin) as those considered in the present dataset. Finally, the terms cited by less than 15% of the panel were eliminated from the list. At the end of the training, the list included 33 descriptors (Table 2).

During the sensory descriptive experiment, the judges had to evaluate monadically the 16 wines (orthonasal and retronasal olfaction) and to rate the intensity of 33 sensory descriptors on linear scales (14 cm); ratings were transformed into scores from 0 to 10. The protocol consisted in 3 repetitions by panelist for the orthonasal olfaction and 2 repetitions by panelist for the retronasal olfaction. Panelists thus performed 5 evaluation sessions (one per week) and started with the 3 orthonasal sessions followed by the 2 retronasal sessions. The presentation order of the wines was counterbalanced according to a Williams Latin square.

**Fig. 1:**
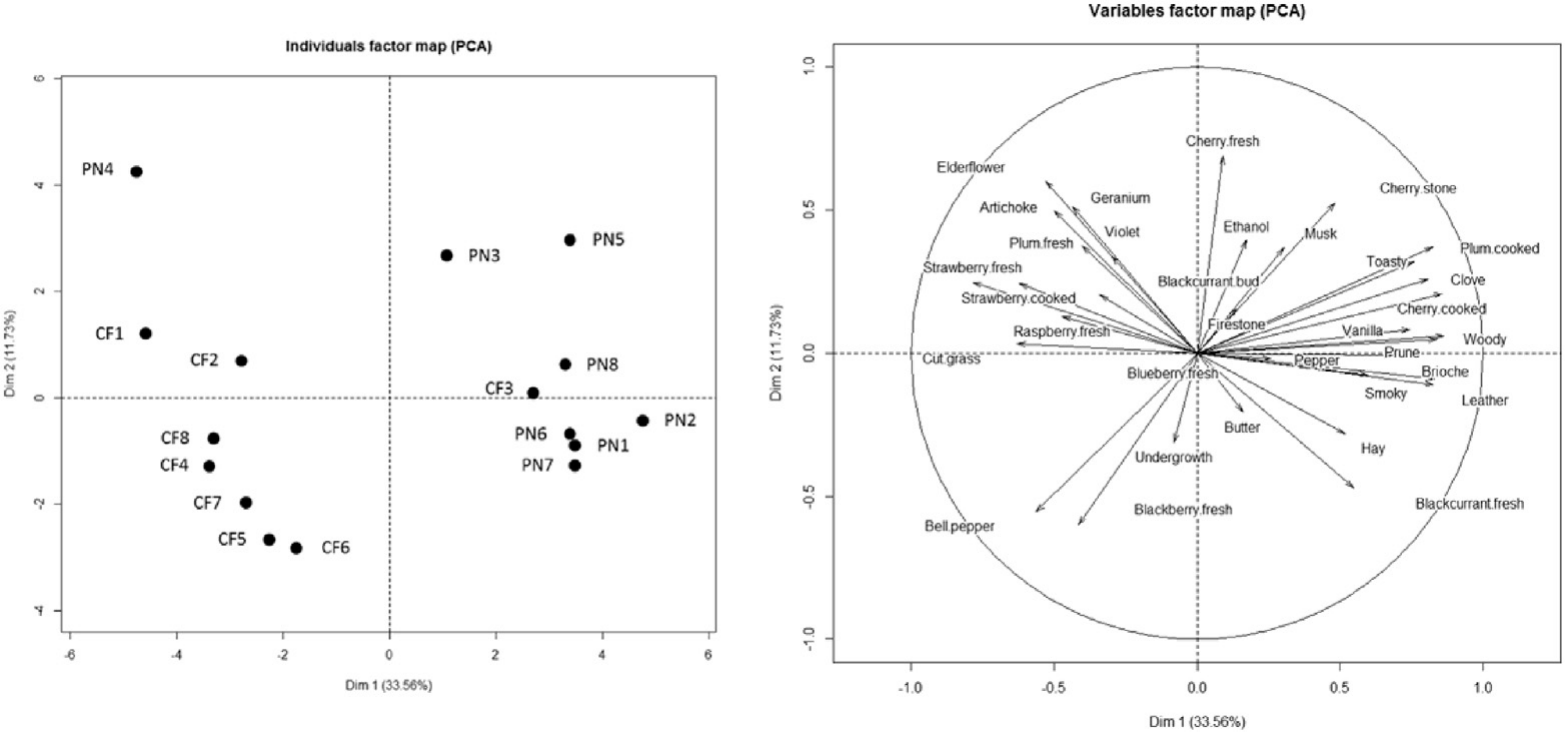
PCA plots, based on the two first dimensions, illustrating the configuration of the 16 wines evaluated using 33 sensory descriptors of orthonasal olfaction. For each sensory descriptor, the rating data were averaged over panelists and repetitions, and standardized (unit scaling).

In order to depict the data collected through orthonasal olfaction, the rating scores were averaged over panelists and repetitions and then submitted to a standardized Principal Components Analysis (PCA) using the R software (version 3.4.0) and the FactoMineR package (version 1.34). The configuration of the 16 wines, as well as the correlations of the sensory descriptors with the two first principal components are shown in Fig. 1.

### 2.3 Volatile organic compounds quantitative analysis

The 16 wines were analyzed by GC-MS to quantify 45 target compounds (see Table 3). These analyses were carried out by a subcontracting external laboratory (ISO 9001 certification, afaq). The concentrations are reported in μg.L^−1^ in the headspace.

Extraction of volatile compounds was performed by Headspace Solid-Phase Micro-Extraction (HS-SPME) following an optimized protocol dedicated to wine volatile organic compounds used in routine by the specialized company. Wine samples were prepared by adding an internal standard, then acidified and salt saturated. A divinylbenzene (DVB)/carboxen (CAR)/polydimethylsiloxane (PDMS) SPME was used for headspace sampling. Extraction time was 60 min at 45 °C. Volatile organic compounds analysis was then performed by GC-MS. The fiber was thermally desorbed in the 250 °C splitless/split inlet of a GC (Shimadzu 2010) coupled with a mass spectrometer (Shimadzu QP2010+). Volatile compounds were separated on a PEG modified column (DB-FFAP 30  m × 0.32 mm × 0.25 μm). Mass spectra were recorded in electron impact mode (70 eV) with a scan/SIM scanning method.

The identification of acetaldehyde, dimethyl sulfide, ethyl acetate, acetic acid, 2-ethylhexan-1-ol, propionic acid and phenol were carried out by comparison with reference mass spectra (WILEY257, NIST, in-house databases). Their quantification was based on an internal calibration by isotopic dilution with ethanal-^13^C2, dimethyl sulfide-d_6_, ethyl acetate-^13^C2, acetic acid-d_4_, 2-ethylhexan-1-ol-d_17_, propionic acid-d_5_ and phenol-d_6_. The identification and quantification of all other compounds was based on a calibration method with these compounds as reference.

One randomly chosen wine sample was analyzed in five replicates in order to estimate the coefficient of variation on each compound, which ranged from 1.8% for phenol to 58.3% for 2-isobutyl-3-methoxypyrazine.

### 2.4 Analysis of wines by GC-MS-O

The 16 wines were analyzed by GC-MS-O at ONIRIS, UMR CNRS 6144 GEPEA Flavor group (Nantes, France).

#### 2.4.1 Extraction methods

The wines were firstly oxygenated by a Venturi aerator, and then 7 mL of wine was poured in a 22 mL vial tightly capped with a Teflon/silicon septum. Volatile compounds from the wine samples were extracted by a representative procedure [8]. Prior to extraction, vials were incubated at 34 °C for 1 h. After that, volatile compounds were extracted by Headspace Solid Phase Micro-Extraction (HS-SPME) with a Car/PDMS fiber (10 mm length, 85 μm film thickness; Supelco, Bellefonte, PA, USA) placed in the headspace of the vial for 10 min at 34 °C.

#### 2.4.2 Chromatographic conditions

The extracts were analyzed by GC (Agilent Technologies 6890N, Wilmington, DE, USA) coupled with a quadripole mass spectrometer (Agilent Technologies, 5973 Network), a FID and a sniffing port (ODP2, Gerstel, Baltimore, MD, USA) to identify odor-active compounds. Volatile compounds were desorbed in the injection port of the GC (T: 260 °C; splitless mode for 5 min) and separated on a DB-Wax column (length: 30 m, internal diameter: 0.25 mm, film thickness: 0.5 μm). Hydrogen was used as carrier gas at constant flow (1 mL.min^−1^). The oven temperature program was set from 50 °C (0 min) to 80 °C at 5 °C min^−1^, from 80 °C to 200 °C at 10 °C min^−1^ and from 200 to 240 °C (4 min) at 20 °C min^−1^. Effluent from the end of the GC column was split 1:1:1 between the MS, the FID (250 °C, air/H_2_ flow: 450/40 mL.min^−1^), and the sniffing port. Peaks were integrated with MSD Chemstation software (Agilent Technologies). Mass spectra were recorded in electron impact mode (70 eV) between 33 and 300 m/z mass range at a scan rate of 2.7 scan s^−1^.

#### 2.4.3 Olfactometry

GC effluent was carried to the sniffing port using a deactivated and uncoated fused silica capillary column, heated to 200 °C. The sniffing port was supplied with humidified air at 40 °C with a flow of 600 mL.min^−1^.

Olfactometry analyses were conducted by 8 experienced judges. Each judge performed one olfactometric analysis for each wine. Therefore, a total of eight GC-MS-O analyses were carried out for each wine. Judges were asked to express their perceptions via the olfactometric software interface [9], representing an aroma wheel made of 56 descriptors and designed for wine analysis. Characteristics of the perceptions were recorded throughout each judge's analysis and data were directly obtained from the olfactometric software. Odorant zones detected by at least 3 out of 8 judges were reported with their Linear Retention Index at apex and their associated odor descriptors.

#### 2.4.4 Odorant compounds identification

The identification of compounds corresponding to each odorant zone was performed by comparing Linear Retention Index (LRI) and mass spectra of detected compounds with those of the databases (Wiley 6.0, NIST and in-house databases), by injection of the standard compounds when available, and by comparison of the odor perceived with those referenced in databases (in house database and The good scents company database [10]). The list of odor-active compounds is reported in Table 4. Compounds non-identified were named after their apex indices number.

## Funding sources

These data were collected as part of the INNOVAROMA research program on wine, which was conducted with financial support from the regional councils of Pays de la Loire, Centre, Bretagne, and Bourgogne (DINOS-AGRALE-12-2011, DINOS-AGRALE-12-2012, PARI-DINOS-2013).

## References

[1] Villière A., Symoneaux R., Roche A., Eslami A., Perrot N., Le Fur Y., Prost C., Courcoux P., Vigneau E., Thomas-Danguin T., Guérin L. (2018). Dataset on the Characterization of the Flavor of Two Red Wine Varieties Using Sensory Descriptive Analysis, Volatile Organic Compounds Quantitative Analysis by GC-MS and Odorant Composition by GC-MS-O.

[2] Vigneau E., Courcoux P., Lefebvre R., Villière A., Symoneaux R. (2015). Regression trees and random forests as a tool for identifying the volatile organic compounds implied in the olfactory perception of wines. 11th Pangborn Sensory Science Symposium, Gothenburg, Sweden, 23-27 August 2015.

[3] Roche A., Perrot N., Chabin T., Villière A., Symoneaux R., Thomas-Danguin T. (2017). In silico modelling to predict the odor profile of food from its molecular composition using experts' knowledge, fuzzy logic and optimization: application on wines. ISOCS/IEEE International Symposium on Olfaction and Electronic Nose (ISOEN), Montreal, QC, 2017.

[4] Loison A., Symoneaux R., Deneulin P., Thomas-Danguin T., Fant C., Guérin L., Le Fur Y. (2015). Exemplarity measurement and estimation of the level of interjudge agreement for two categories of French red wines. Food Qual. Prefer..

[5] ISO 3591:1977/Sensory analysis -- Apparatus -- Wine-tasting glass.

[6] Lawless H.T., Heymann H. (1998). Sensory Evaluation of Food: Practices and Principals. Food Science Texts Series.

[7] Noble A.C., Arnold R.A., Buechsenstein J., Leach E.J., Schmidt J.O., Stern P.M. (1987). Modification of a standardized system of wine aroma terminology. Am. J. Enol. Vitic..

[8] Villière A., Arvisenet G., Lethuaut L., Prost C., Sérot T. (2012). Selection of a representative extraction method for the analysis of odourant volatile composition of French cider by GC-MS-O and GC × GC-TOF-MS. Food Chem..

[9] Villière A., Le Roy S., Fillonneau C., Guillet F., Falquerho H., Boussely S., Prost C. (2015). Evaluation of aroma profile differences between sué, sautéed, and pan-fried onions using an innovative olfactometric approach. Flavour.

[10] The Good Scents Company, (n.d.). http://www.thegoodscentscompany.com/search2.html (Accessed 3 October 2017).

